# “My Tummy Tells Me” Cognitions, Barriers and Supports of Parents and School-Age Children for Appropriate Portion Sizes

**DOI:** 10.3390/nu10081040

**Published:** 2018-08-08

**Authors:** Kaitlyn M. Eck, Colleen L. Delaney, Miriam P. Leary, Oluremi A. Famodou, Melissa D. Olfert, Karla P. Shelnutt, Carol Byrd-Bredbenner

**Affiliations:** 1 Department of Nutritional Sciences, Rutgers University, 26 Nichol Avenue, New Brunswick, NJ 08901, USA; colleenldelaney90@gmail.com (C.L.D.); bredbenner@sebs.rutgers.edu (C.B.-B.); 2 Division of Animal and Nutritional Sciences, West Virginia University, 1194 Evansdale Dr. G28, West Virginia University, Morgantown, WV 26506, USA; miriampearman@gmail.com (M.P.L.); Melissa.Olfert@mail.wvu.edu (M.D.O.); 3 Department of Family, Youth, and Community Sciences, University of Florida, Gainesville, FL 32611, USA; oluremifamodu@tcomn.com (O.A.F.); kpagan@ufl.edu (K.P.S.)

**Keywords:** parent, child, portions, focus groups, theory

## Abstract

Larger portion sizes have increased in tandem with the rise in obesity. Elucidation of the cognitions of children and parents related to portion size is needed to inform the development of effective obesity prevention programs. This study examined cognitions of parents (*n* = 36) and their school-age children (6 to 11 years; *n* = 35) related to portion sizes via focus group discussions. Parents and children believed controlling portion sizes promoted health and weight control. Some parents felt controlling portions was unnecessary, particularly if kids were a healthy weight because kids can self-regulate intake. Barriers to serving appropriate portions identified by parents focused largely on kids getting enough, rather than too much, to eat. Parents also identified lack of knowledge of age-appropriate portions as a barrier. Facilitators of portion control cited by parents included purchasing pre-portioned products and using small containers to serve food. Children relied on cues from parents (e.g., amount of food parent served them) and internal hunger/satiety cues to regulate intake but found it difficult to avoid overeating highly palatable foods, at restaurants, and when others were overeating. Results suggest obesity prevention interventions should aim to improve portion sizes cognitions, barrier management, and use of facilitators, in families with school-age children.

## 1. Introduction

The size of portions individuals typically eat have increased in tandem with the rise in obesity [1,2,3]. A commonly cited factor contributing to today’s larger portion sizes is the changes in restaurant portion sizes over the past decades. In fact, restaurant portions are up to eight times larger than serving sizes set by the U.S. Department of Agriculture (USDA) and the Food and Drug Administration (FDA) [4,5]. These hefty restaurant portions have distorted perceptions of the actual amount of food needed for good health and have thereby “normalized” outsized portions to the extent that even portions served at home are larger than needed [4]. 

Larger portion sizes result in increased daily energy intake for both children and adults, suggesting their potential for increasing weight [6,7]. When children are served large portions, they increase the size of their bites and overall amount of food consumed [7]. Instead, if children are allowed to serve themselves, they typically consume a smaller amount of food [7]. Teaching parents of young children about age-appropriate portion sizes promotes healthy energy intake in children in part by improving parents’ ability to estimate appropriate portions [6].

Healthy children are born with an innate ability to self-regulate their dietary intake to match their needs [8]. Unfortunately, well-meaning parents can teach children to distrust their internal hunger and fullness cues by serving portions that exceed children’s needs which, in turn, prompts children to overeat [9]. Parents may serve portions larger than children need because they are not aware of portion sizes appropriate for children [10,11]. Parents may push children to eat more than they need out of a lack of understanding of children’s ability to self-regulate [12], desire to not waste food [13], fear the child is not eating enough to grow normally [14], or for reasons of convenience for the parent, particularly when parents are stressed or the child is eating slowly [13,14,15]. Tactics that pressure kids to eat more than needed include offering rewards or punishment [7,16,17].

Parents can foster children’s ability to self-regulate by allowing kids to serve themselves at mealtime and snacktime in accordance with their personal level of hunger [7,18]. The generally accepted responsibility of parents regarding child feeding is that they, as food gatekeepers, are responsible for the purchase and preparation of healthy foods from which children can choose. It is the responsibility of children to decide what and how much they will eat [19].

Parents report limited knowledge of appropriately sized portions [11,20] and many parents engage in feeding practices that are associated with poor outcomes, such as development of behaviors linked to undesirable weight gain [14]. For instance, children whose parents pressure them to eat are more likely to overeat, eat in the absence of hunger, experience loss of control over eating, become hyper responsive to food cues (i.e., the sight or smell of food), and have a tendency toward emotional eating [7,17,21]. In addition, some evidence indicates that parents can learn to effectively coach children who have lost their ability to self-regulate, to regain use of internal hunger and satiety cues [22]. Clearly, interventions are needed to help parents learn to promote and serve portion sizes matched to children’s need as well as use healthy child feeding practices. 

The overarching framework of Social Cognitive Theory (SCT) is based on reciprocal determinism, that is, the concept that individuals are both influenced by and can influence their environment [23,24,25]. SCT highlights the ability of individuals to change their environment, within the home for example, to facilitate behavior change. SCT focuses both on individual and group behavior change, which makes this theory an ideal fit for guiding behavior change within families [23,24,25]. Constructs of SCT including collective efficacy (beliefs about the group’s ability to make a change) and observational learning (learning behaviors through peer modeling) are particularly relevant for use in interventions targeting the family environment [23,24,25]. Additionally, understanding individuals’ outcome expectations related to behavior change, as well as their self-identified barriers to and facilitators for performing behaviors can help interventionists provide audience-specific guidance that promotes behavior change [23,24,25].

To develop a program that will enable parents to adjust portion sizes and feeding practices to support optimal child health and prevent obesity, a clearer understanding of parent cognitions (e.g., attitudes, perceived barriers, facilitators) vis-à-vis portion size is needed. School-age children rely on their parents to make some of their food-related decisions, but kids also consume about a one-third of their meals away from home [12] with this proportion increasing into the teen years [26,27]. Consequently, elucidation of the cognitions of children related to portion size also is needed. Thus, this study aimed to use key constructs from SCT [23,24,25] as a guide to understanding parent and child cognitions regarding portion size choices with the ultimate goal of using findings to inform the development of materials designed to improve the healthfulness of portion sizes selected by parents and school-age children. 

## 2. Materials and Methods

The Institutional Review Boards at the authors’ universities granted permission to conduct this study. Informed consent was obtained from parents for themselves as well as child participants. Verbal assent was given by child participants prior to the start of data collection. 

### 2.1. Sample

Parents who had at least one school-age child (aged 6 to 11 years) were recruited to participate in focus groups held in New Jersey (NJ), West Virginia (WV), and Florida (FL). Printed and electronic recruitment announcements were circulated via community centers, schools, worksites, houses of worship, and other venues frequented by parents. These announcements asked parents to join discussions about their home and lifestyle practices and strategies they could use to help them raise happier, healthier families. These announcements also informed parents of the opportunity to enroll their school-age children in a similar group discussion. Recruitment notices were in both English and Spanish and indicated parent focus groups would last approximately one hour, and child focus groups would be about 30 min in length. Announcements also indicated parents would receive $25 and children $15 for their participation. Parents and children who participated in the focus groups were not necessarily related to one another.

### 2.2. Instrument

Parent and child participants answered a brief questionnaire that gathered demographic information (e.g., age, highest education level) before engaging in semi-structured focus group discussions. Parents also were asked to estimate how often they allowed their children to decide quantities to eat at mealtime. The focus group discussion items were grounded in SCT, a behavior change theory emphasizing the bi-directional interaction between individuals and the environment. [23,24,25], addressing key constructs associated with behavior development and change. These constructs included attitudes toward portion size, barriers to appropriately sized portions, and facilitators for serving appropriately sized portions.

Each focus group was conducted by a team of two researchers. All focus group researchers completed formal training and practice sessions to ensure skillful, uniform conduct and management of focus groups across all data collection locations. One team member served as the focus group moderator and adhered closely to the focus group moderator guide, which was developed and refined using standard procedures and guidelines [28,29]. The second researcher took comprehensive notes and transcribed them within 48 h. Note transcription also included translation of Spanish focus group data into English. The moderator who conducted the focus group checked all transcribed notes to ensure completeness, accuracy, and clarity. Finally, both researchers conferred to reach full agreement on the content of the transcribed notes.

Parent focus groups were conducted in the participants’ primary language (i.e., either English or Spanish). All child focus groups were in English because all were fluent in English. Young children (i.e., ages 6 to 9 years) participated in groups separate from those of more mature, older children (i.e., ages 9 to 11 years). To promote privacy and limit distractions, data collection areas were limited to focus group researchers and participants.

### 2.3. Data Analysis

Descriptive statistics for demographic data were generated via SPSS version 21.0 (Chicago, IL, USA). Three researchers independently applied standard qualitative content analysis procedures to extract themes from the focus group data [30,31]. Content analysis procedures were applied because they produce impartial, methodically derived descriptions of the data themes [32,33]. Three trained researchers reviewed the data independently and identified trends and themes using Excel spreadsheets to manage the data. Content analysis results were compared and discussed to reach consensus across researchers. Data were analyzed continuously throughout data collection to recognize when the point of information redundancy was reached, and data collection could conclude [30,34].

## 3. Results

There was a total of 36 parents (93% female) of school-aged children (ages 6 to 11 years) who each participated in 1 of the 14 focus group discussions held with parents. Parents had an average age of 39.82 ± 6.87 stand deviation (SD) years and had 2.39 ± 1.05 SD children under the age of 18 living at home. Most parents (67%) had at least some post-secondary education or higher. Two-thirds completed the focus groups in English vs. 33% in Spanish. The number of focus groups and participants was comparable across states (FL: 4 groups, *n* = 13; NJ: 4 groups, *n* = 12; WV: 5 groups, *n* = 12). Participants in each focus group were unique (no parents participated in more than one focus group).

A total of 35 children (43% female) participated in 1 of the 12 focus group discussions held with children. Children had an average age of 8.00 ± 1.46 SD years. The children had a mean of 1.00 ± 0.89 SD siblings younger and 1.29 ± 1.84 SD siblings older than them. The number of child focus groups and participants per state (FL: 3 groups, *n* = 11; NJ: 5 groups, *n* = 12; WV: 4 groups, *n* = 12) was distributed almost evenly across states. No child participated in more than one focus group.

### 3.1. Parent Focus Groups 

Questionnaire results indicated parents let their children decide how much to eat at mealtimes about half the days in a week (i.e., 3.53 ± 1.00 SD days/week). Although sample sizes were small, it is important to note that English-speaking parents allowed children to decide amounts to eat significantly (*p* < 0.001) more often than Spanish-speaking parents (3.92 ± 0.93 SD vs. 2.75 ± 0.62 SD days/week, respectively). A comparison of frequency of allowing children to decide amounts to eat revealed similar results across parent education level and geographic locations. 

#### 3.1.1. Parents’ Attitudes toward Appropriately Sized Food Portions

Most parents felt it was important for them to serve their children appropriately sized portions because they wanted to “monitor what (the child) eats” so the child had “good health and not be obese” or “chubby”. Parents controlled portions because they did not want their child to feel “yucky” or “make himself sick” by eating too much. One parent noted the importance of portion size control; however, she indicated that her child “is body conscious, so it worries me to talk about this”.

Some parents indicated it was not especially important to concern themselves with serving their children age-appropriate amounts of food because “kids are the best judge of how much they need to eat, as long as what we give them is healthy”. These parents felt children will eat “when they are hungry” and “eat what they want” because they are able to “self-regulate” and “understand hunger cues”. Others agreed it was important to let the “body figure out how much it needs”, especially if kids are physically active, and to let children “make their own healthy choices and be responsible”. One mom reported that she lets her children “serve themselves” and “does not make them eat anything they don’t want to” because as a child, she was forced to “clean her plate” which impaired her ability to self-regulate and caused her to become overweight. 

Others who did not concern themselves with serving children age-appropriate portion sizes commented, “My kids are overeaters, but I don’t limit how much they can have. If they are hungry, they can eat”. Similarly, others indicated, “I just want him to be satisfied”, but qualified this with “but if I saw that he puts on weight, I would worry” and “my kids eat when they are hungry; they eat what they want. If it becomes a problem, then I’ll go from there”. Another pointed out that it is “hard to know the difference between body type and unhealthy” weight gain.

When asked how they determined how much their children should eat, some parents thought it was a “guessing game”. Portion sizes for children were “based on how much they normally eat”, by “the size and age of my child”, and by “serving them a little” at a time. Others reported using various aids to determine portion size, including a chart, their hand, small plate size, plates with dividers, serving 1 tablespoon of food per year of age, and following advice from the child’s physician. A few parents stated that “we need to be better informed of how much they need to eat” because “if I do not control it now, then it becomes (more) difficult” as kids get older. 

Many parents realized that the amount of food they ate affected the amount their children ate. Parents remarked that their children “notice how much is on my plate constantly” and “imitate some of the things we do; as parents, we set an example” of how much to eat or what to eat. “Well I think that kids might watch me and say, ‘my mom eats a lot and she is huge’. I don’t think that I am going to eat like her because I don’t want to end up like my mom”. Others reported modeling positive behavior, but did not “consciously say ‘look kids’” to encourage children to mimic their behavior of eating appropriately sized food portions. A few parents believed that their children did not “pay any attention to what I am eating” and “role modeling doesn’t seem to affect kids often”, commenting that “I don’t think that if I had 5 pieces of pizza, my kid would also eat 5”. Parents felt their children “understand that the hunger of an adult and a child isn’t the same thing” because “adults need more food” so kids “base their portion around the amount of hunger that they have”, not the amount adults are eating. Another influencer of eating appropriately sized portions was siblings because “they complain if one gets more or less”, while peers “don’t really have an influence” on portion sizes selected. 

The type of food influenced the importance parents placed on portion control. “For things they like, like junk food, they won’t realize how much they ate” if it was not portioned out. One mother stated she “cares less about the calories” and has more “worries about the sugar” in foods, which motivated her to regulate the portion size of sugary foods. Parents report that calorie-rich foods such as “candy has to be controlled” and “ice cream and cookies are portioned, but things like yogurt and healthy foods” are less of a concern. Similarly, parents “wouldn’t really monitor a meal like dinner, but I would monitor dessert”.

Lastly, several parents believed it has become easier now that their children are school-aged to keep portion sizes appropriate due to having a “routine with meals”. As children have more “structured meals”, they are eating smaller portions of snacks.

#### 3.1.2. Parents’ Perceived Barriers to Serving Appropriately Sized Food Portions

Parent responses to barriers to serving appropriately sized food portions indicated they were mostly concerned that kids were not getting enough to eat at mealtimes. Many reported children did not eat sufficient amounts of food during mealtimes due to “snacking” between meals, being picky eaters, appetite depression during growth plateaus, and hectic schedules that interfered with eating at home. Having enough money was cited as an obstacle to providing enough food because “organic and protein are more expensive”. A few parents noted that “nothing really prevents me” from serving kids enough food because kids “they have to eat what they’re given”. There was little mention of barriers to preventing oversized portions, although a few offered “if they want more, try to talk them through the decision”, “make them wait to see if they are actually still hungry” before serving more, and “it is better to err on the size of too small—better to have him decide if he wants more or not.”

Another perceived barrier parents reported was a lack of knowledge about serving their children appropriately sized portions. They were uncertain about “what the specific guidelines are right now” for appropriate serving sizes and felt a “need to know what the proper portion is”. “Anything that you could give us on portions would be helpful; we really want to know so that we can feed our children the exact amount that they should be eating”. This reported lack of knowledge was similar to reports made by parents of preschool children who also noted lack of knowledge about age-appropriate portions and expresses an eagerness to learn more about healthy portions [35]. 

#### 3.1.3. Parents’ Strategies for Overcoming Barriers to Serving Appropriately Sized Food Portions

To ensure children eat enough, parents suggested getting the children “involved in the process” of food preparation and “taking them to the supermarket”. Parents also suggested “talking to them about their foods”, and “motivating them” to eat enough for “their own-well-being”. Others encouraged kids to eat enough by modeling healthy eating behaviors.

Parents had an array of ideas for helping children keep portions from being too large. For instance, they suggested buying “pre-portioned” foods. “Well, you can buy the large bag of chips and then it is hard to eat one portion, but you can buy a package of small bags of chips instead of the large one and have a smaller portion for one person”. Other recommendations were to exert control over larger portions by individually wrapping each cookie or serving portions from larger containers. Another portion control strategy was to serve food in “little containers” or on smaller plates. More covert methods of controlling food intake were to have “more fruit and vegetables on the plate” to limit space for less unhealthy choices, “hide or lock food away”, and have a “squeaky pantry door, so I know if they are snacking too much”. Role modeling was another strategy, “I try to get them to mirror my behaviors, such as limiting salad dressing”. Additionally, parents felt that it would be helpful to know the “portions that should be given for each age group”, perhaps by learning from another parent “who has gone through an experience” related to portion control or asking a professional for help and advice. 

Overall, the discussion about portion size was thought provoking for many participants. “It is very important to think about these questions that you have asked us today”.

### 3.2. Children’s Focus Groups

#### 3.2.1. Children’s Attitudes toward Appropriately Sized Food Portions

Most children indicated that eating enough, but not too much food was important, so they could “grow”, “stay healthy”, “not get sick”, and not “be too skinny or too fat”. Several commented that eating enough was important because they exercise (“It is important because I have to play sports”, “If you are exercising a lot, you should eat a lot of food”). Kids also reported it was important to avoid eating “a lot of calories” and “too much food” to prevent a “stomachache” and “feeling sick”. Some realized a balance was important, “having too much food is a bad thing because the body could hurt” but if they did “not have enough (they) could be hungry”.

Children agreed that their parents thought it was “really important” for kids to eat enough because they “need to grow”, it “makes you healthy”, and “they want you to grow up to be strong”. Children also indicated that parents did not want them to overeat because “they don’t want you to get sick from too much junk food or too much food”.

#### 3.2.2. Children’s Decisions on Amount to Eat

Children stated that they know it is time to eat “by your brain telling you” and “tummy growling”. They determined how much to eat using physical signals (“eat until you’re full”, “my tummy tells me how much to eat”), parent guidance (“my mom tells me how much to eat”, parent “puts food on my plate”), past experiences with food, and by eating “not a lot of food, but not a little bit of food”. Kids stop eating when their “stomach starts hurting”, “can’t eat anymore”, plate is empty, or parents let them leave the table.

Many children felt parents “know how much you should eat” because “they’re older so they’ve experienced it”, and “their (own) parents have told them in the past” the right amount to eat. Others commented that parents “know servings and read the package and that’s what they give you”. On the contrary, some children expressed that parents do not measure the amount of food and “just grab whatever” to “fill up your plate” or “give me food and I eat until I don’t want to eat anymore”. A few indicated they did not know how parents decided how much kids should eat.

#### 3.2.3. Children’s Perceived Barriers to Not Overeating

Children said that “very delicious stuff” and “things I really like–pizza, watermelon, ice cream” made them overeat as did “see(ing) something that is really tasty”. They also noted that inactivity promoted eating (“I eat too much because I am bored”, “If I am watching TV, I have to go to the cabinet and take a snack”). Others reported that getting hungry leads to eating too much (“I overeat because I’m starving”). One child mentioned that “eating mostly outside (of the home) at restaurants” was a barrier to keeping portion size under control.

#### 3.2.4. Children’s Strategies for Overcoming Barriers to Not Overeating

Children reported that they relied on parents to help them not overeat. (“They guard you from eating too much–mom tells you not to eat too much”. “My dad—if he thinks we’re eating a lot, he will tell us to wait and see if we feel full and if we’re still hungry later (we can have more).) Others remarked, “They tell me if I eat too much food” and “I ask my parents, and if I am full I don’t eat more; I stop”. Some relied on intuitive cues, “my tummy tells me (to stop eating)”. A few children indicated that they “look at what parents eat and eat less”.

## 4. Discussion

The aim of this study was to identify the cognitions and behaviors of parents and school-aged children related to portion size. The discussion below and Table 1 suggests how these findings can be used to inform future obesity prevention programs grounded in SCT [23,24,25]. 

### 4.1. Outcome Expectations

Focus group discussion findings revealed that parents were unsure of the importance of regulating portion size for their children. Many believed children had an innate ability to regulate their intake using internal hunger and satiety cues, and thus, did not feel concerned about serving age-appropriate portions. This finding suggests that obesity prevention programs could aim to help parents learn age-appropriate serving sizes, encourage them to actively compare child intake with recommendations, and protect children’s weight and health by offering guidance to children when portions eaten stray significantly above or below recommendations.

Most parents recognized the link between age-appropriate portions and healthy weight and good health, yet some felt they did not need to be concerned about children’s portion sizes if children had healthy weights. Using child weight as a method for determining whether to invoke portion size control is worrisome given that parents of overweight or obese children often misclassify their children’s weights as being healthy [36]. Furthermore, the importance of lifelong portion size control is important because eating habits established in childhood track into adulthood [37] and that overweight and obese children are likely to be overweight and obese adults [38,39]. This focus group finding suggests a need to help parents be proactive in teaching children portion size control to manage weight rather than using it as a weight loss method after children have gained excess weight.

The benefits of appropriately sized portions identified by children were similar to those reported by parents. Children understood that consuming “not too much and not too little” was important to their health and body weight. However, there was an emphasis on the need to consume enough (larger portions) to support exercise. Children’s calorie needs typically are not greatly influenced by physical activity because most children in the United States engage in physical activity less than one hour per day. Research indicates that adults are not able to accurately estimate the amount of energy expended during exercise [40,41], and overestimates of energy expenditure result in overeating [42]. Parents also overestimate children’s physical activity levels [43,44] and, as a result, are likely to overestimate calories needed to compensate for exercise, which may lead them to encourage children to over eat. Active children (engaging in physical activity equal to walking 3 miles/day at 3 to 4 miles per hour) need only about 200 to 400 more calories than sedentary or moderately active children [45]. Helping parents and children understand how calories burned in exercise are more modest than many realize [40,41,43,44] could enable them to better balance dietary intake with energy expenditure in exercise, and concomitantly better match portion size to needs. 

### 4.2. Self-Efficacy

Parents were less willing to allow children to self-regulate portion sizes of calorie-rich foods, such as candy and other desserts, because children were reported to tend to overconsume these foods. Parents offered a limited array of ideas for controlling portion sizes of these foods, with most methods being restrictions delivered in an authoritarian, rather than instructive, manner. Food restrictions imposed by parents are linked with higher body mass index (BMI) in children [14]. General parenting style also is associated with BMI with children of authoritative parents having healthier BMIs than children whose parents have authoritarian and permissive parenting styles [14]. This difference may be because the feeding practices employed by authoritarian and permissive parents are less likely to support the development of healthy self-regulation skills in children than the feeding strategies used by authoritative parents [46]. Therefore, future nutrition interventions could benefit families by incorporating instruction on alternative methods for portion control that build children’s own self-regulation skills.

### 4.3. Facilitation

Strategies used by parents to determine how much to serve children varied greatly, and few parents reported knowing how much to feed their children. Many parents identified this lack of knowledge as a major barrier to serving age-appropriate portions. Parent knowledge of appropriate portion size is inversely associated with healthy child weight status [11,12]. Parents in this study expressed a willingness to learn and an interest in educational materials that would help them understand age-appropriate portion sizes. Thus, it is important to improve parents’ knowledge of appropriately sized portions for kids, as well as themselves, while also increasing their self-efficacy for engaging in portion control by providing tools and resources, such as measuring cups and spoons. 

Some children reported using internal hunger and satiety cues to determine how much to eat whereas others relied on their parents (e.g., how much parents served them) to decide amounts to eat. Kids expected parents to provide guidance on how much to eat and some reported they need permission before getting second helpings at meals. Depending on parents to determine portion size can be troublesome as children mature and increasingly eat away from their families [47] because kids will not be equipped to make portion size decisions independently. Furthermore, if children are accustomed to determining how much to eat by the amount parents serve them, it is possible children will overeat when served large restaurant portions. Empowering children to tune into their own hunger and satiety cues may be useful in promoting healthy portion sizes. Nutrition interventions should encourage parents to build children’s self-efficacy for regulating their own intake by allowing kids to serve themselves at mealtime. Another challenge is that parents who decide portion sizes may have a distorted idea of what constitutes an appropriately sized portion [48,49]. Learning to choose appropriate serving sizes for themselves is an important self-management skill children need to gain. Future nutrition education programs could teach these skills directly to children as well as build parent abilities and confidence for teaching their children about portion control.

Children repeatedly mentioned tuning in to internal satiety signals to determine when to stop eating. However, in several cases, the signal mentioned was my “stomach starts hurting”—a sign of overeating rather than satiety. Thus, children would benefit from learning how to accurately identify hunger and satiety cues and better use these cues to regulate food intake. Improving parents’ self-efficacy for coaching children to use these internal cues could aid in child development of these skills.

The barriers to portion control reported by parents focused mainly on getting children to eat enough food at meals. Few parents were concerned with scaling down portion sizes to ensure kids were not eating too much at mealtime. Parents seemed to not recognize that large portion sizes of foods they considered to be healthy, such as meat and milk, also could have negative consequences. Future nutrition interventions should address the importance of controlling portions of all foods, including those that are healthy foods. Snacking was cited as a key reason children ate portions at meals that were smaller than parents believed kids should eat. As a result, parent strategies for controlling portions focused mostly on snacks foods, which tended to be energy-dense and nutrient empty. With the rise in snacking occasions in school-age children, it will be important for future nutrition education programs to highlight appropriate serving sizes at snack time, healthy snack options, how snack choice and serving size needs to be put in context with the calorie and nutrient content of mealtime foods, and the importance of snack timing vis-à-vis mealtime to ensure adequate mealtime intake. Parents displayed limited ideas for controlling overeating thereby suggesting that incorporating simple ideas for controlling portion sizes and controlling intake into future nutrition intervention programs may be useful. 

Children reported that a trigger to overeating was extreme hunger. Providing parents and kids with strategies for improved timing of meals and snacks to manage hunger may improve the effectiveness of future obesity prevention programs focused on improving portion sizes.

### 4.4. Observational Learning

Modeling was another strategy parents employed to help regulate their children’s dietary intake. Parents recognized that that the amount of food they ate affected the amount their children ate and leveraged their role as a model to encourage their kids to mimic their healthy behaviors related to portion size. Appropriate portion sizes for adults are often not appropriate for children [45], and some parents noted that children were able to distinguish between parents and child needs. Kids also described modeling their plate after their parents, taking slightly smaller amounts to meet their needs. However, parents may not realize that their modeling of large portion sizes now could affect their child’s future behaviors. Children may come to recognize the portions parents are consuming as ideal adult portions and adapt those behaviors as they grow into adulthood. Enhancing parents’ understanding of the effect of role modeling on their children’s behavior now and in the future may enhance parents’ abilities to model behaviors that support children’s abilities to regulate their own portion sizes as they grow into adulthood.

Parents noted that portion sizes eaten by siblings influenced children. However, parents did not believe peers influenced children’s portion sizes. Although this may be the case now, the influence of peers is likely to increase as children age. Subjective norms have been shown to influence adolescent eating behaviors [50,51], and the size of portions consumed by individuals can be influenced by eating partners, particularly when the partners feel they belong to the same social group [52,53].

One parent expressed concerned about discussing portion control with her body conscious child for fear of triggering disordered eating behaviors. As the age of onset of eating disorders is found to be decreasing [54], the Academy for Eating Disorders recommends that nutrition programs deliver nutrition information in a manner that promotes health and well-being rather than weight loss [55]. Additionally, nutrition programs should empower parents to talk to their children about body image [55].

### 4.5. Demographic Differences

Survey results revealed that English-speaking parents allowed children to decide amounts to eat more often than Spanish-speaking parents. This is similar to the results of a study that reported Latino mothers who spoke Spanish were significantly more likely to pressure their children to eat and control the type of food their children consumed than Latino mothers who spoke English as well as non-Hispanic English speakers [56]. This suggests that cultural variations related to portion size should be addressed in future interventions. 

Survey results reveled that there was no significant relationship between parent education level and the frequency of parents allowing children to decide how much to eat. Previous research has shown an association between parent education level and child feeding practices. For example, a study of parents in the UK found that highly educated parents were significantly more likely to use food rewards and punishments than their less educated counterparts but found no difference in control over eating, emotional feeding, or encouragement to eat [57]. Other studies have reported that educated mothers are more likely to exert control over feeding, encourage healthy eating and are less likely to engage in emotional feeding practices [58,59].

## 5. Conclusions

Parents’ perceptions of the importance of healthy portions sizes were mixed, and parents appeared to be more concerned with serving portions that were too small rather than too large, highlighting the need to improve parents’ outcome expectations. The main facilitators identified by parents included using pre-portioned packages and small serving containers, and parents identified lack of knowledge as their greatest barrier to serving appropriate portions. These findings indicate a need for nutrition education programs that aim to improve parent’s knowledge of age-appropriate portions while providing strategies for overcoming barriers to serving healthy portions to facilitate behavior change. Additionally, school-age children continue to rely on their parents to make many food-related decisions for them, but as these children begin to spend more time outside of the home, their personal responsibilities related to food increase. Therefore, future interventions should aim to build children’s confidence in regulating their own portion sizes. While this study focuses on changes that can be made on the individual or family level, large scale environmental and policy changes related to portions served at restaurants, or the size of pre-packaged food containers could further aid these changes. 

This is one of the first studies to qualitatively examine both parent and child cognitions related to portion size. The small sample size in this study may be seen as a limitation; however, the qualitative focus group data are rich and data saturation was reached prior to termination of data collection. Understanding cognitions of both parents and children related to portions is critical for the development of future nutrition related interventions aiming to improve health-related behaviors and prevent childhood obesity. Findings from this study and the suggestions outlined in Table 1 can be used to inform the development of future nutrition interventions aiming to improve healthy portions sizes in families with school-aged children. 

## Figures and Tables

**Table 1:** Recommendations for Interventions Promoting Age-Appropriate Portion Sizes in Families with School-Age Children.

Social Cognitive Theory Construct	Recommendations for Future Interventions Promoting Age-Appropriate Portion Sizes
Facilitation	Provide parents with guidelines for age-appropriate serving sizes.
Self-efficacy	Build parent confidence in their ability to compare child intake with age-appropriate serving size recommendations and offer guidance to children when portions are too large or small.
Outcome expectations	Expand parent views of portion control as a weight management tool rather than a weight loss method.
Facilitation	Promote parent feeding styles that allow children to build their own self-regulation skills.
Facilitation	Enhance parent and child knowledge of energy expenditure in exercise to enable them to balance energy intake with expenditure.
Facilitation/Self-efficacy	Provide parents with tools and resources to help them feel confident in their ability to serve age-appropriate portions.
Self-efficacy	Build children’s confidence in their ability to serve their own meals and regulate intake using internal hunger and satiety cues.
Self-efficacy	Build parent confidence in teaching kids about appropriately sized portions.
Self-efficacy	Build parent confidence in coaching children to use their internal hunger and satiety cues.
Outcome Expectation	Expand parent perceptions of the importance of portion control to include healthy foods.
Facilitation	Provide parents with ideas for simple and effective ways to control portion sizes and minimize overeating.
Outcome Expectations	Enhance parent understanding of the effect of role modeling on children’s behavior now and in the future.
Self-efficacy	Empower parents to effectively talk with their children about body image.
Facilitation	Address cultural variations in parent feeding behaviors.

## References

[1] Hales C.M., Fryar C.D., Carroll M.D., Freedman D.S., Ogden C.L. (2018). Trends in obesity and severe obesity prevalence in us youth and adults by sex and age, 2007–2008 to 2015–2016. JAMA.

[2] Livingstone M.B.E., Pourshahidi L.K. (2014). Portion size and obesity. Adv. Nutr..

[3] Piernas C., Popkin B.M. (2011). Food portion patterns and trends among us children and the relationship to total eating occasion size, 1977–2006. J. Nutr..

[4] Steenhuis I.H.M., Vermeer W.M. (2009). Portion size: Review and framework for interventions. Int. J. Behav. Nutr. Phys. Act..

[5] Young L.R., Nestle M. (2003). Expanding portion sizes in the U.S. marketplace: Implications for nutrition counseling. J. Am. Diet. Assoc..

[6] Small L., Lane H., Vaughan L., Melnyk B., McBurnett D. (2013). A systematic review of the evidence: The effects of portion size manipulation with children and portion education/training interventions on dietary intake with adults. Worldviews Evid. Based Nurs..

[7] Birch L.L., Fisher J.O., Davison K.K. (2003). Learning to overeat: Maternal use of restrictive feeding practices promotes girls’ eating in the absence of hunger. Am. J. Clin. Nutr..

[8] Fox M.K., Devaney B., Reidy K., Razafindrakoto C., Ziegler P. (2006). Relationship between portion size and energy intake among infants and toddlers: Evidence of self-regulation. J. Am. Diet. Assoc..

[9] Rhee K. (2008). Childhood overweight and the relationship between parent behaviors, parenting style, and family functioning. Ann. Am. Acad. Pol. Soc. Sci..

[10] McCrickerd K., Forde C.G. (2016). Parents, portions and potential distortions: Unpicking children’s meal size. Nutr. Bull..

[11] Croker H., Sweetman C., Cooke L. (2009). Mothers’ views on portion sizes for children. J. Hum. Nutr. Diet..

[12] Poti J.M., Popkin B.M. (2011). Trends in energy intake among us children by eating location and food source, 1977–2006. J. Am. Diet. Assoc..

[13] Momin S.R., Chung K.R., Olson B.H. (2014). A qualitative study to understand positive and negative child feeding behaviors of immigrant Asian Indian mothers in the US. Matern. Child. Health J..

[14] Shloim N., Edelson L.R., Martin N., Hetherington M.M. (2015). Parenting styles, feeding styles, feeding practices, and weight status in 4–12 year-old children: A systematic review of the literature. Front. Psychol..

[15] Berge J.M., Tate A., Trofholz A., Fertig A.R., Miner M., Crow S., Neumark-Sztainer D. (2017). Momentary parental stress and food-related parenting practices. Pediatrics.

[16] Matheson B.E., Camacho C., Peterson C.B., Rhee K.E., Rydell S.A., Zucker N.L., Boutelle K.N. (2015). The relationship between parent feeding styles and general parenting with loss of control eating in treatment-seeking overweight and obese children. Int. J. Eat. Disord..

[17] Farrow C.V., Haycraft E., Blissett J.M. (2015). Teaching our children when to eat: How parental feeding practices inform the development of emotional eating—A longitudinal experimental design. Am. J. Clin. Nutr..

[18] Kraak V.A., Liverman C.T., Koplan J.P. (2005). Preventing Childhood Obesity: Health in the Balance.

[19] Satter E. Raise a Healthy Child who Is a Joy to Feed: Follow the Division of Responsibility in Feeding. www.ellynsatterinstitute.org/how-to-feed/the-division-of-responsibility-in-feeding/.

[20] Sherry B., McDivitt J., Birch L.L., Cook F.H., Sanders S., Prish J.L., Francis L.A., Scanlon K.S. (2004). Attitudes, practices, and concerns about child feeding and child weight status among socioeconomically diverse white, Hispanic, and African-American mothers. J. Am. Diet. Assoc..

[21] Vaughn A., Ward D., Fisher J., Faith M., Hughes S., Kremers S., Musher-Eizenman D., O’Connor T., Patrick H., Power T. (2015). Fundamental constructs in food parenting practices: A content map to guide future research. Nutr. Rev..

[22] Johnson S.L. (2000). Improving preschoolers’ self-regulation of energy intake. Pediatrics.

[23] Bandura A. (2004). Health promotion by social cognitive means. Health Educ. Behav..

[24] Bandura A. (1977). A Social Learning Theory.

[25] Kelder S., Hoelscher D., Perry C., Glanz K., Rimer B., Viswanath K. (2015). How individuals, environments, and health behavior interact. Health Behavior, Theory, Research, and Practice.

[26] Poti J.M., Duffey K.J., Popkin B.M. (2013). The association of fast food consumption with poor dietary outcomes and obesity among children: Is it the fast food or the remainder of the diet?. Am. J. Clin. Nutr..

[27] French S.A., Story M., Neumark-Sztainer D., Fulkerson J.A., Hannan P. (2001). Fast food restaurant use among adolescents: Associations with nutrient intake, food choices and behavioral and psychosocial variables. Int. J. Obes. Relat. Metab. Disord..

[28] Rollnick S., Mason P., Butler C. (1999). Health Behavior Change: A Guide for Practitioners.

[29] McLafferty I. (2004). Focus group interviews as a data collection strategy. J. Adv. Nurs..

[30] Harris J.E., Gleason P.M., Sheean P.M., Boushey C., Beto J.A., Bruemmer B. (2009). An introduction to qualitative research for food and nutrition professionals. J. Am. Diet Assoc..

[31] Miles M., Huberman A. (1994). Qualitative Data Analysis.

[32] Berelson B. (1971). Content Analysis in Community Research.

[33] Krippendorff K. (1980). Content Analysis: An Introduction to Its Methodology.

[34] Sandelowski M. (1995). Sample size in qualitative research. Res. Nurs. Health.

[35] Martin-Biggers J., Spaccarotella K., Hongu N., Alleman G., Worobey J., Byrd-Bredbenner C. (2015). Translating it into real life: A qualitative study of the cognitions, barriers and supports for key obesogenic behaviors of parents of preschoolers. BMC Public Health.

[36] Tompkins C.L., Seablom M., Brock D.W. (2015). Parental perception of child’s body weight: A systematic review. J. Child Fam. Stud..

[37] Movassagh E.Z., Baxter-Jones A.D.G., Kontulainen S., Whiting S.J., Vatanparast H. (2017). Tracking dietary patterns over 20 years from childhood through adolescence into young adulthood: The Saskatchewan pediatric bone mineral accrual study. Nutrients.

[38] Hales C., Carroll M., Fryar C., Ogden C. (2017). Prevalence of Obesity Among Adults and Youth: United States, 2015–2016. Nchs Data Brief, No. 288.

[39] Simmonds M., Llewellyn A., Owen C.G., Woolacott N. (2016). Predicting adult obesity from childhood obesity: A systematic review and meta-analysis. Obes. Rev..

[40] Brown R.E., Canning K.L., Fung M., Jiandani D., Riddell M.C., Macpherson A.K., Kuk J.L. (2016). Calorie estimation in adults differing in body weight class and weight loss status. Med. Sci. Sport Exerc..

[41] Willbond S.M., Laviolette M.A., Duval K., Doucet E. (2010). Normal weight men and women overestimate exercise energy expenditure. J. Sports Med. Phys. Fit..

[42] McCaig D.C., Hawkins L.A., Rogers P.J. (2016). License to eat: Information on energy expended during exercise affects subsequent energy intake. Appetite.

[43] Kesten J.M., Jago R., Sebire S.J., Edwards M.J., Pool L., Zahra J., Thompson J.L. (2015). Understanding the accuracy of parental perceptions of child physical activity: A mixed methods analysis. J. Phys. Act. Health.

[44] Lau J., Engelen L., Bundy A. (2013). Parents’ perceptions of children’s physical activity compared on two electronic diaries. Pediatr. Exerc. Sci..

[45] Kerksick C., Fox E. (2016). Sports Nutrition Needs for Child and Adolescent Athletes.

[46] Kiefner-Burmeister A., Hoffmann D., Zbur S., Musher-Eizenman D. (2016). Implementation of parental feeding practices: Does parenting style matter?. Public Health Nutr..

[47] McClain A.C., Ayala G.X., Sotres-Alvarez D., Siega-Riz A.M., Kaplan R.C., Gellman M.D., Gallo L.C., Van Horn L., Daviglus M.L., Perera M.J. (2018). Frequency of intake and type of away-from- home foods consumed are associated with diet quality in the hispanic community health study/study of latinos (hchs/sol). J. Nutr..

[48] Smith L.P., Ng S.W., Popkin B.M. (2013). Trends in us home food preparation and consumption: Analysis of national nutrition surveys and time use studies from 1965–1966 to 2007–2008. Nutr. J..

[49] Schwartz J., Byrd-Bredbenner C. (2006). The ability of young adults to estimate portion size and calorie content. Top. Clin. Nutr..

[50] Stok F.M., de Vet E., de Wit J.B., Luszczynska A., Safron M., de Ridder D.T. (2015). The proof is in the eating: Subjective peer norms are associated with adolescents’ eating behaviour. Public Health Nutr..

[51] Cruwys T., Bevelander K.E., Hermans R.C. (2015). Social modeling of eating: A review of when and why social influence affects food intake and choice. Appetite.

[52] Vartanian L.R., Sokol N., Herman C.P., Polivy J. (2013). Social models provide a norm of appropriate food intake for young women. PLoS ONE.

[53] Higgs S., Thomas J. (2016). Social influences on eating. Curr. Opin. Behav. Sci..

[54] Favaro A., Caregaro L., Tenconi E., Bosello R., Santonastaso P. (2009). Time trends in age at onset of anorexia nervosa and bulimia nervosa. J. Clin. Psychol..

[55] Academy for Eating Disorders Guidelines for Childhood Obesity Prevention Programs. www.aedweb.org/advocate/press-releases/position-statements/guidelines-childhood-obesity.

[56] Seth J.G., Evans A.E., Harris K.K., Loyo J.J., Ray T.C., Spaulding C., Gottlieb N.H. (2007). Preschooler feeding practices and beliefs: Differences among spanish- and english-speaking wic clients. Fam. Community Health.

[57] Clark H.R., Goyder E., Bissell P., Blank L., Walters S.J., Peters J. (2008). A pilot survey of socio-economic differences in child-feeding behaviours among parents of primary-school children. Public Health Nutr..

[58] Saxton J., Carnell S., Van Jaarsveld C.H., Wardle J. (2009). Maternal education is associated with feeding style. J. Am. Diet. Assoc..

[59] Monge-Rojas R., Smith-Castro V., Colon-Ramos U., Garita-Arce C., Sánchez-López M., Chinnock A. (2010). Parental feeding styles and adolescents’ healthy eating habits. Structure and correlates of a costa rican questionnaire. Appetite.

